# The National MD-PhD Program Outcomes Study: career paths followed by Black and Hispanic graduates

**DOI:** 10.1172/jci.insight.178248

**Published:** 2024-05-08

**Authors:** Myles H. Akabas, Lawrence F. Brass

**Affiliations:** 1 Neuroscience and Medicine, Albert Einstein College of Medicine, Bronx, New York, USA.; 2 Medicine and Pharmacology, University of Pennsylvania Perelman School of Medicine, Philadelphia, Pennsylvania, USA.

## Abstract

Previous studies on attrition from MD-PhD programs have shown that students who self-identify as Black are more likely to withdraw before graduating than Hispanic students and students not from groups underrepresented in medicine (non-UIM). Here, we analyzed data collected for the National MD-PhD Program Outcomes Study, a national effort to track the careers of over 10,000 individuals who have graduated from MD-PhD programs over the past 60 years. On average, Black trainees took slightly longer to graduate, were less likely to choose careers in academia, and were more likely to enter nonacademic clinical practice; although, none of these differences were large. Black graduates were also more likely to choose careers in surgery or internal medicine, or entirely forego residency, and less likely to choose pediatrics, pathology, or neurology. Among those in academia, average research effort rates self-reported by Black, Hispanic, and non-UIM alumni were indistinguishable, as were rates of obtaining research grants and mentored training awards. However, the proportion of Black and Hispanic alumni who reported having NIH research grants was lower than that of non-UIM alumni, and the NIH career development to research project grant (K-to-R) conversion rate was lower for Black alumni. We propose that the reasons for these differences reflect experiences before, during, and after training and, therefore, conclude with action items that address each of these stages.

## Introduction

MD-PhD programs were originally established by a small number of medical schools in the 1950s and 1960s to provide rigorous research training for future clinicians (1, 2). The earliest programs were small and primarily populated by White men (3). In the decades that followed, program numbers and size have increased, as has program diversity. More women have been admitted, as have members of groups historically underrepresented in medicine and science (UIM). However, while the number of women entering MD-PhD programs has increased considerably (and recently reached parity with that of men), entry of UIM individuals has increased minimally (3).

Attrition in MD-PhD programs varies among programs, but overall it is relatively low. Most applicants who begin a program stay in and finish it (4). However, a 2014 study of students matriculating from 1995 to 2000 suggested that as a group UIM students were more likely to be dismissed or to withdraw from MD-PhD programs than non-UIM students (5). The differences were neither substantial nor statistically significant: the odds ratio (95% confidence interval) for either withdrawal or dismissal was 1.32 (0.97–1.79) relative to White students. However, a more recent study of MD-PhD students matriculating from 2004 to 2012 found that Black students were 50% more likely to leave the program and graduate with an MD only compared with White students (adjusted odds ratio, 1.50; 95% confidence interval, 1.04–2.16) and 83% more likely to leave medical school entirely (odds ratio, 1.83; 95% confidence interval, 1.06–3.16). In contrast, attrition rates for Hispanic students were indistinguishable from those of White students (6).

Given these reports, we asked whether career choices and career outcomes for Black and Hispanic students who graduated from MD-PhD programs are distinguishable from those of their non-UIM peers. To answer this question, we have taken advantage of an existing data set on 50-plus years of program graduates that was collected in 2015 for the National MD-PhD Program Outcomes Study, a joint project of the MD-PhD Section of the Association of American Medical Colleges (AAMC), the leadership of the individual MD-PhD programs, and the research staff of the AAMC. Eighty programs participated, representing 92% of then-current trainees and 44 of the 45 programs that were receiving National Institute of General Medical Sciences (NIGMS) Medical Scientist Training Program (MSTP) T32 training grant support at the time. In that study, 10,591 graduates were identified. Surveys were completed by 64% of them (76% of those with valid email addresses) and combined with data on all alumni from the AAMC Student Record System, Faculty Roster, and Graduate Medical Education-Track databases. This produced a deidentified data set substantially larger than anything available previously or since (2).

In a prior report based on data from the national study, we considered outcomes for aggregate UIM alumni and compared them with those for non-UIM alumni (3). Here, we have broken out data on individuals who identify as Black or Hispanic to look at key outcomes, such as time to degree, primary workplace, research effort for those in academia, success at obtaining research grants from any source, and NIH K-to-R conversion rates. Because of evidence that the choice of a clinical specialty affects the likelihood of having a research-focused career as a physician-scientist (7), we also compared residency choices made by Black, Hispanic, and non-UIM MD-PhD program alumni. We did not include individuals who identify as American Indian or Alaska Native or Native Hawaiian or Other Pacific Islander due to the very small number of individuals in each group who have completed MD-PhD programs. Because of the small number of UIM graduates prior to 2000, we limited key parts of our analysis to the 4,390 survey respondents who graduated from 2000 to 2014.

The results show that Black alumni take a bit longer to graduate then Hispanic and non-UIM alumni; are less likely to choose careers in pediatrics, pathology, and neurology; and are more likely to choose surgery or internal medicine. Black alumni, but not Hispanic alumni, are also less likely to have chosen careers in academia and more likely to have entered nonacademic clinical practice. Among those who chose careers in academia, rates of research effort reported by Black, Hispanic, and non-UIM alumni were similar, as were the overall rates of obtaining research grants and, for recent graduates, mentored training awards. However, the proportion of both Black and Hispanic alumni who reported having NIH research grants was lower than that of non-UIM alumni, and the fraction of Black alumni who obtained NIH research grants after previously being awarded a mentored career award (K grant) was lower than that for Hispanic and non-UIM individuals.

## Results

### Time to degree.

In the previous analysis of the 2015 National MD-PhD Programs Outcomes study data, all members of UIM groups defined by the NIH were pooled to calculate time to MD-PhD program completion (2, 3). Here, we reanalyzed the data, separating Black and Hispanic alumni. Because there were few (*n* = 77) UIM alumni prior to 2000 and because of the steady increase in average time to degree that has occurred over the past 50 years (2), we limited our initial analysis to alumni who graduated from 2000 to 2014. For Black alumni, the time to degree in this period was 8.55 ± 1.26 years (*n* = 206); for Hispanic alumni, the average was 8.23 ± 1.09 years (*n* = 187); and for non-UIM alumni, the average time to degree was 8.19 ± 1.09 years (*n* = 3,933; mean ± SD). By 1-way ANOVA, the time to degree for Black alumni was significantly different than that for Hispanic (*P* = 0.01) and non-UIM alumni (*P* < 0.0001). For Hispanic alumni, the time to degree was not significantly different than that for non-UIM alumni.

There has been a general upward creep in time to degree for MD-PhD program graduates (2); therefore, we also performed a secondary analysis limiting the data set to the most recent 5-year cohort of graduates (2010–2014). The same general trend was observed: the average time to degree was 8.52 ± 1.31 years for Black alumni (*n* = 89), 8.34 ± 1.16 years for Hispanic alumni (*n* = 94), and 8.25 ± 1.04 years for non-UIM alumni (*n* = 1,631; mean ± SD). However, the differences between the groups were slightly smaller and no longer significant by 1-way ANOVA.

### Choice of clinical specialty for residency training.

**Figure 1:**
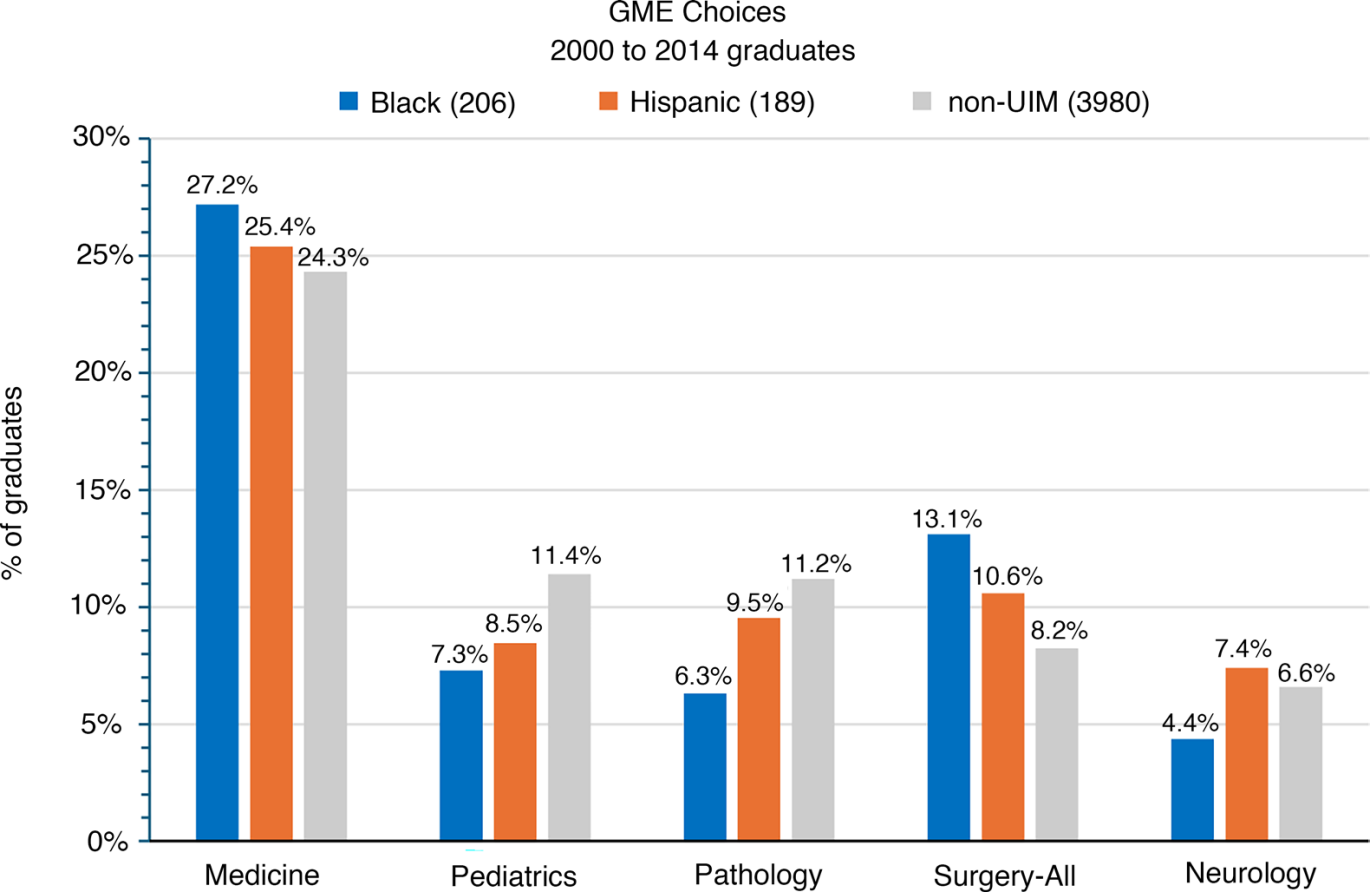
Graduate medical education choices made by Black, Hispanic, and non-UIM MD-PhD program alumni who graduated between 2000 and 2014. The 5 most popular clinical fields, including the combined result for all surgery disciplines, are shown. The complete data set, which includes the individual surgery specialties and alumni who chose not to do additional clinical training, is shown in Table 1. GME, graduate medical education.

For alumni who graduated between 2000 and 2014, we compared the distribution of residency fields for postgraduate training chosen by 206 Black alumni, 187 Hispanic alumni, and 3,980 non-UIM alumni (Figure 1 and Table 1). Among the 5 most popular fields (internal medicine, pediatrics, pathology, surgery, and neurology), Black alumni were less likely to choose pediatrics, pathology, and neurology than Hispanic or non-UIM alumni and were more likely to choose surgery and internal medicine. Note that “surgery” as used here includes general surgery and the surgical specialties listed in Table 1. Hispanic alumni were more likely to choose surgery and neurology than non-UIM alumni. Non-UIM alumni were more likely to choose pediatrics and pathology than either Black or Hispanic alumni. However, in all cases, these differences even when proportionately large, amount to just a few percentage points. Finally, for reasons that were not captured by the survey, Black alumni were more likely to report choosing to not do a residency than either Hispanic or non-UIM alumni (Table 1).

### Primary workplace.

**Figure 2:**
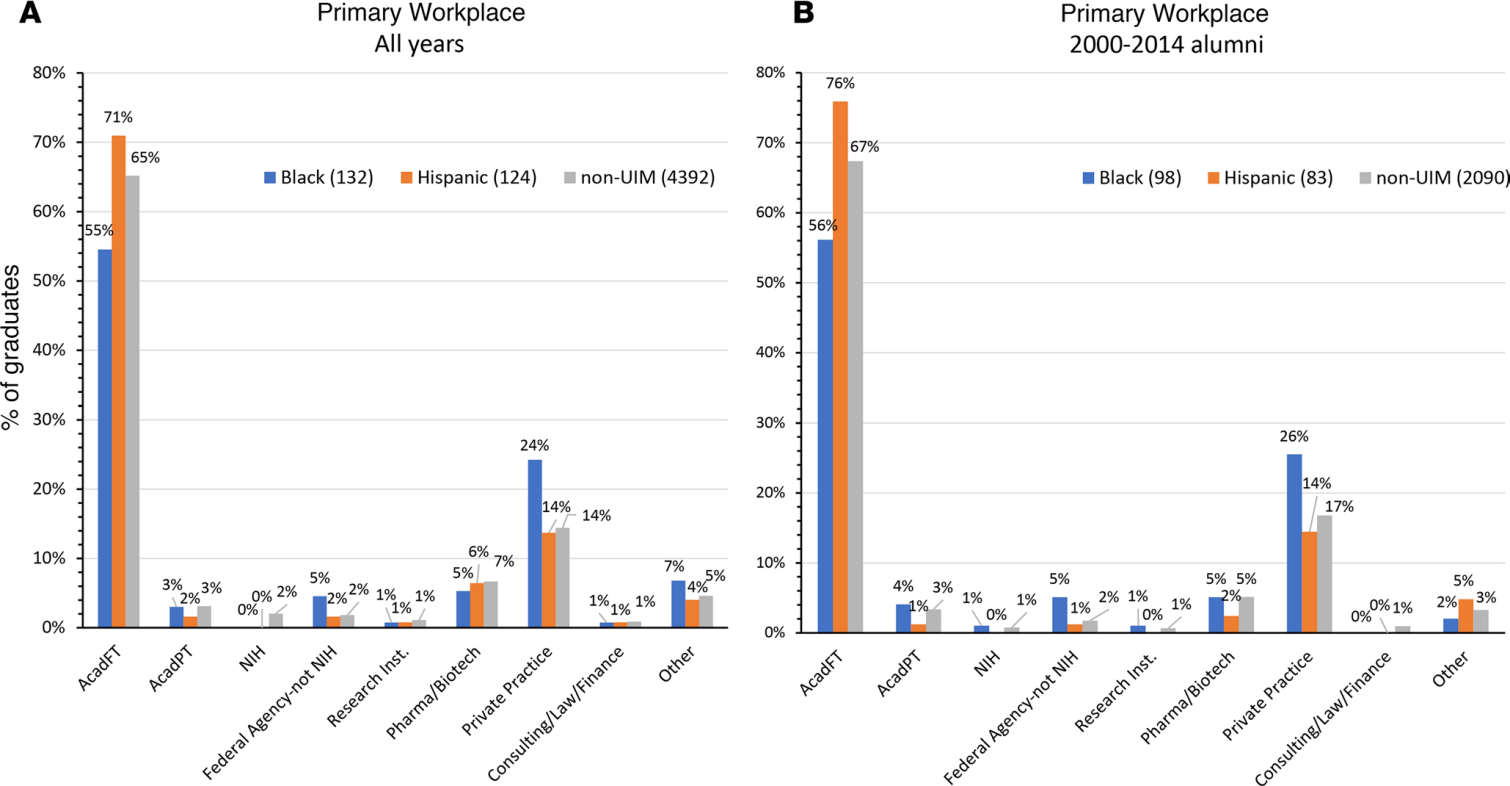
Primary workplace for MD-PhD program alumni. (**A**) All MD-PhD program alumni who completed postgraduate training and answered the survey. “All years” refers to years prior to 1975 through 2014. (**B**) Alumni who graduated from 2000 to 2014. AcadFT, academia full time; AcadPT, academia part time.

Figure 2A shows the current workplace for 132 Black alumni, 124 Hispanic alumni, and 4,392 non-UIM alumni who had completed all phases of postgraduate training at the time that the survey data were collected. Categories available in the survey were academia full time, academia part time, NIH, a federal agency other than the NIH (e.g., the CDC or FDA), a research institute, industry (biotech or pharmaceutical), nonacademic clinical practice (i.e., private practice), consulting/law/finance, and other. Workplace distribution was similar between Hispanic and non-UIM alumni. Black alumni were less frequently in academia full time (55% vs. 65%) and more frequently in private practice (24% vs. 14%). Similar differences among alumni groups were present when the comparison was limited to those who graduated from 2000 to 2014 (Figure 2B).

### Research effort for alumni in academia full time.

**Figure 3:**
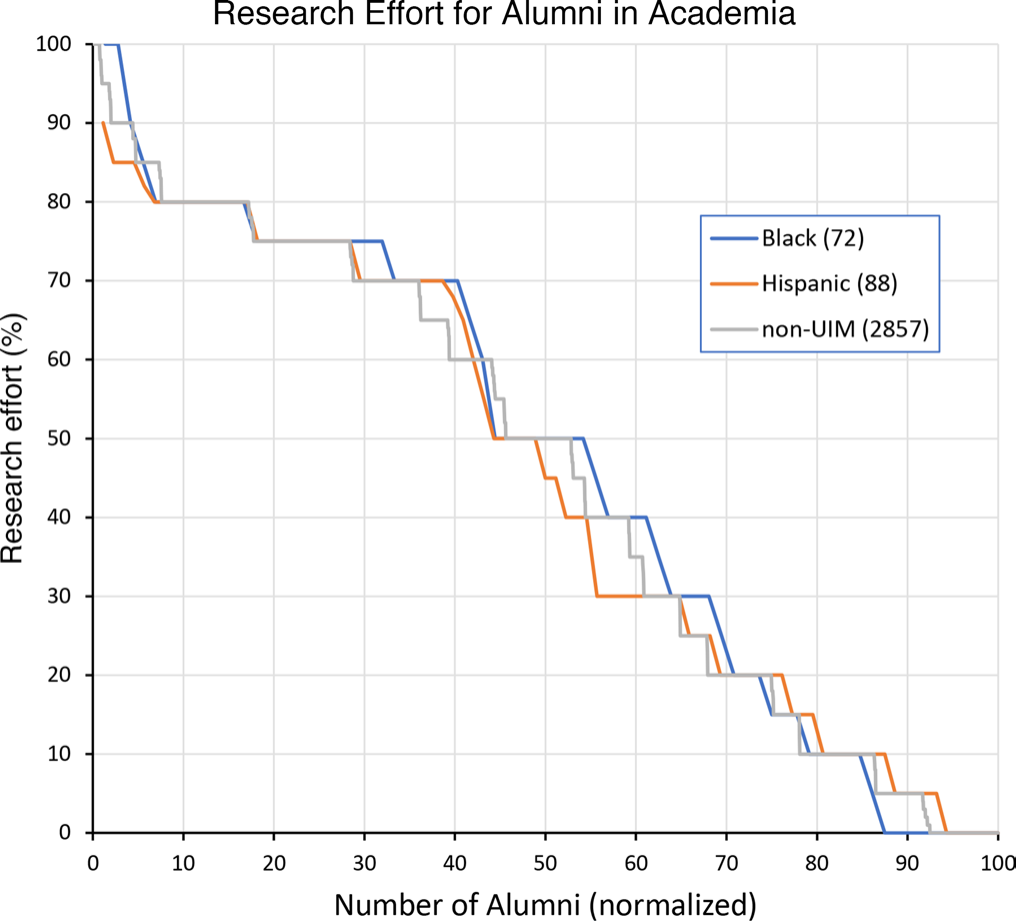
Research effort for MD-PhD program alumni in academia. Self-reported data on research effort as a percentage of total professional effort for all survey respondents who had completed training and reported their current position as academia full time. The *x* axis has been normalized so that 0–100 reflects the rank-ordered distribution for 72 Black alumni, 88 Hispanic alumni, and 2,857 non-UIM alumni. The average research effort for the 3 groups was 46%, 45%, and 46%, respectively.

Alumni who had completed postgraduate training at the time of the survey were asked to indicate how they divided their time among research, clinical care, teaching, and administration. In the survey instructions, teaching was defined as including classroom lectures, small-group preceptorships, and teaching in the clinical setting. The rank-ordered distribution of self-reported research effort for 72 Black alumni, 88 Hispanic alumni, and 2,857 non-UIM alumni working in academia full time is shown in Figure 3. There were no differences between groups in either the distribution of effort or average research effort (46%, 45%, and 46%).

### Research funding for alumni in academia.

**Figure 4:**
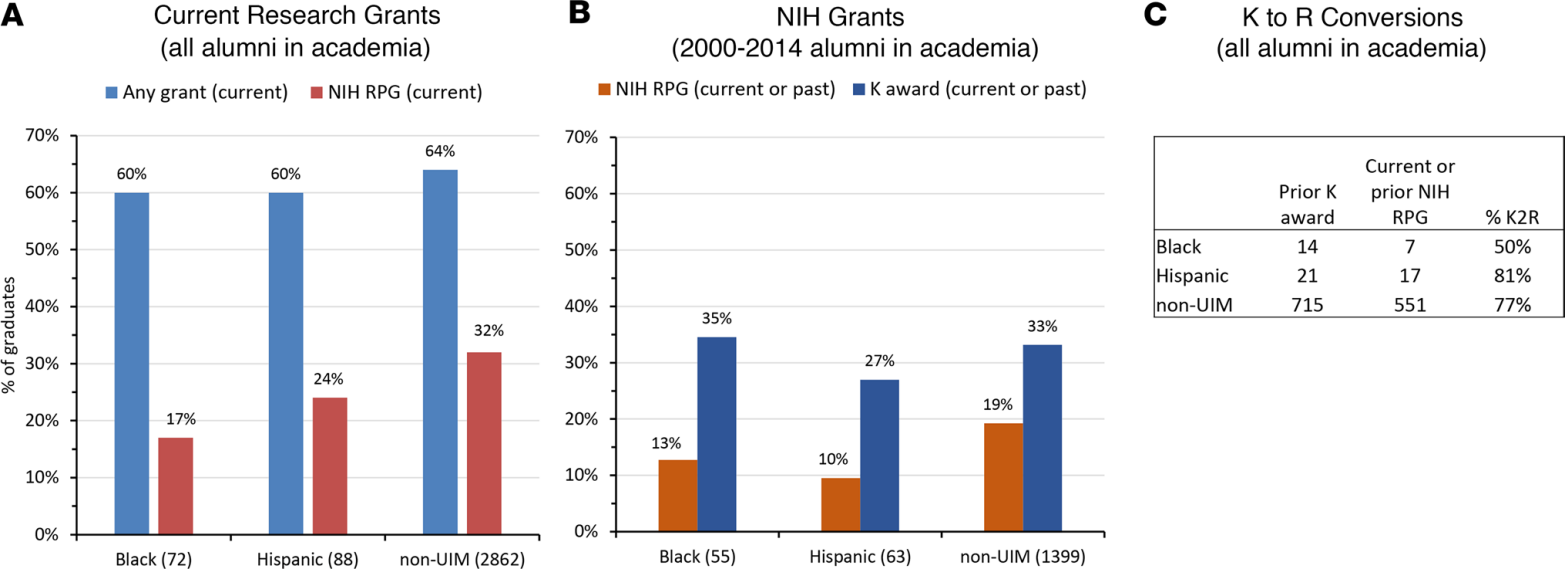
Research funding for MD-PhD program alumni in academia. Self-reported data from the National MD-PhD Outcomes Survey. (**A**) Research grants currently held by all alumni who were in academia at the time of the survey. The blue bars show grants from any source, including the NIH. The red bars refer to NIH research program grants (RPGs). (**B**) Previous and current NIH RPG data (red bars) and mentored K award data (blue bars) for graduates from 2000 to 2014 who had completed postgraduate training and were employed in academia full time when the survey was conducted. (**C**) Summarized data from all alumni on K awards and NIH research grants held by the same individual. K2R refers to the fraction of alumni in each group who held an NIH RPG after having previously held a K award.

Alumni were also asked for information about research grants for which they were the principal investigator, including the source of the grant. Overall funding rates for current grants from any source were about 60% in all 3 groups, but the NIH funding rate was nearly twice as high for those in the non-UIM group than for Black alumni (Figure 4A). Funding rates for Hispanic alumni fell in between those for non-UIM and Black alumni. Figure 4B shows both previous and current NIH research project grants and mentored training awards for individuals who graduated from 2000 to 2014. K award rates were similar for all 3 groups. If anything, the comparatively small number of Black MD-PhD alumni had a higher K award rate than everyone else. However, the K-to-R conversion rate was smaller for Black alumni than for either the Hispanic or non-UIM alumni (Figure 4C). NIH research project grants were less prevalent in this cohort than in program graduates as a whole. The rate for Black and Hispanic alumni was less than that for White alumni.

## Discussion

A recent report suggested that Black individuals are more likely to withdraw from MD-PhD programs (6). Here, we used data from the National MD-PhD Programs Outcomes study to ask whether the career outcomes of the Black and Hispanic students who remained in their program and graduated are the same as those for non-UIM graduates. The results show that Black alumni, but not Hispanic alumni, take a bit longer to graduate with both doctorates, are less likely to choose careers in academia, and are more likely to enter nonacademic clinical practice than non-UIM alumni. Compared with their non-UIM counterparts, Black alumni were less likely to have chosen careers in pediatrics, pathology, or neurology and more likely to have chosen internal medicine or surgery or to forego residency entirely. Notably, however, none of these differences are large. Among those who chose a career in academia, the aggregate time spent on research reported by Black, Hispanic and non-UIM alumni was indistinguishable. So were the overall rates of obtaining research grants and, for recent graduates, the rates of obtaining mentored training awards. Notably, however, fewer Black and Hispanic alumni reported having NIH research grants, and the proportion of those who obtained mentored K awards and subsequently obtained research project grants (the NIH K-to-R conversion rate) was lower for Black alumni than for other alumni groups. This latter observation is consistent with prior work showing that Black applicants are less likely to receive NIH R01 grants (8).

### Strengths and weaknesses.

Before considering the reasons for these differences and what, if anything, should be done about them, it is worth considering the strengths and the limitations of the data set upon which the conclusions are based. The greatest strength of the National MD-PhD Programs Outcomes Study is the large number of participating programs, the large number of alumni who provided data, the high survey response rate, and the availability of AAMC databases that included information about MD-PhD program alumni who subsequently became faculty at US medical schools. As there were so few UIM graduates prior to 2000, we compared data for all graduation years and then for just the most recent 15-year period (2000–2014). The results were similar. Differences in racial and ethnic representation have also been noted by others using a wider swath of medicine than just physician-scientist careers (9–11).

If the size of the data set is a strength, the reliance on self-reported data for research effort and research funding is a weakness. Unfortunately, although NIH databases include information on NIH grants, there are no equivalent resources for research funding from industry, professional societies, and foundations. Bias related to self-reporting may account for why the K-to-R conversion rates shown in Table 1 are higher than the conversion rates reported in studies that include all recipients of K awards and not just those who graduated from an MD-PhD program (12). Finally, it should be noted that the survey data were collected in 2015 and were, therefore, 8 years old at the time that this manuscript was written. What has happened to older alumni in the years since and what choices are being made by newer graduates will, we hope, become the subject of a planned follow-up study. Hopefully, that study will also provide greater insight into the reasons behind the choices made by MD-PhD students and alumni. Absent that information, we can only draw inferences, including those discussed below.

### Lessons learned.

Although we began this study with a focus on Black and Hispanic alumni, the data from the National MD-PhD Outcomes study along with other recent efforts to understand obstacles on the physician-scientist career path offer useful lessons that apply to overlapping sets of individuals from disadvantaged backgrounds. First and foremost, the demographic data show that efforts to recruit and sustain a more diverse physician-scientist workforce have been only modestly successful. The number of applicants to MD-PhD programs has been static at 1,800–2,000 for more than a decade (2). During this period, there has been a pronounced increase in the number of women applicants but, despite national efforts, little increase in individuals from other underrepresented groups (3, 13). The reasons are undoubtedly numerous but likely include shortfalls in awareness, encouragement, and support. Others have shown that first-generation college graduates are as likely as others to consider MD-PhD training but are 30% less likely to apply and matriculate and that students with large loan burdens are less likely to consider MD-PhD training (14).

A second lesson is that applying to MD-PhD programs has become ever more time consuming and expensive, both of which likely have a disproportionate effect on individuals who have fewer resources from which to draw. Successful applicants are expected to have spent years in college and afterward working in research settings where they may gain valuable experience but limited compensation (15). Tolerance of deferred compensation and avoidance of undergraduate debt are likely to be two of the reasons that most MD-PhD program applicants come from upper socioeconomic strata. The need for awareness and encouragement helps explain why many applicants come from homes in which one or both parents hold advanced degrees (16). Application costs are considerable (17), hitting applicants long before tuition waivers and stipends kick in to help them. Once accepted, predoctoral stipends for MD-PhD students can be insufficient to cover the local cost of living, especially for trainees with prior debts and/or responsibilities for others. Robust support and encouragement from family, friends, and MD-PhD program leaders plays an important role in the ability to persist on a career path that commonly includes a decade or two of deferred compensation.

If the first two lessons are about recruiting and applying, both of which are within the purview of MD-PhD programs, a third lesson has to do with career choices made by trainees before and after they graduate. Sooner or later, every trainee will be faced with important decisions, including how much time they will devote to research, which clinical discipline (if any) they will choose for postgraduate training, how persistent they will be in the endless competition for research funding, and whether they will choose to work in settings in which their research training will be essential. These are choices whose consequences linger for years and affect the likelihood that the graduate will be able to sustain a career as a physician-scientist. To what extent does growing up in an underrepresented group affect these decisions? Groups underrepresented in medicine and science are no more homogeneous than any other group of applicants. Some individuals may also be socioeconomically disadvantaged; others may not. Although every well-coached applicant to an MD-PhD program talks about their commitment to a research-focused career, to what extent do the higher salaries that can be commanded by physicians in procedure-oriented fields make those fields more attractive to one group of MD-PhD program students than another, perhaps leading them to choose a clinical discipline that will leave them less time for research? Differences in research productivity and research funding can follow from these decisions. In the larger frame, it doesn’t matter if, as we observed here, Black MD-PhD students are a bit more likely to become internists or surgeons and a bit less likely to become pediatricians, neurologists, or pathologists. It does matter if they start out to become a physician-scientist and end up with little or no time for research.

### Actions to consider.

**Figure 5:**
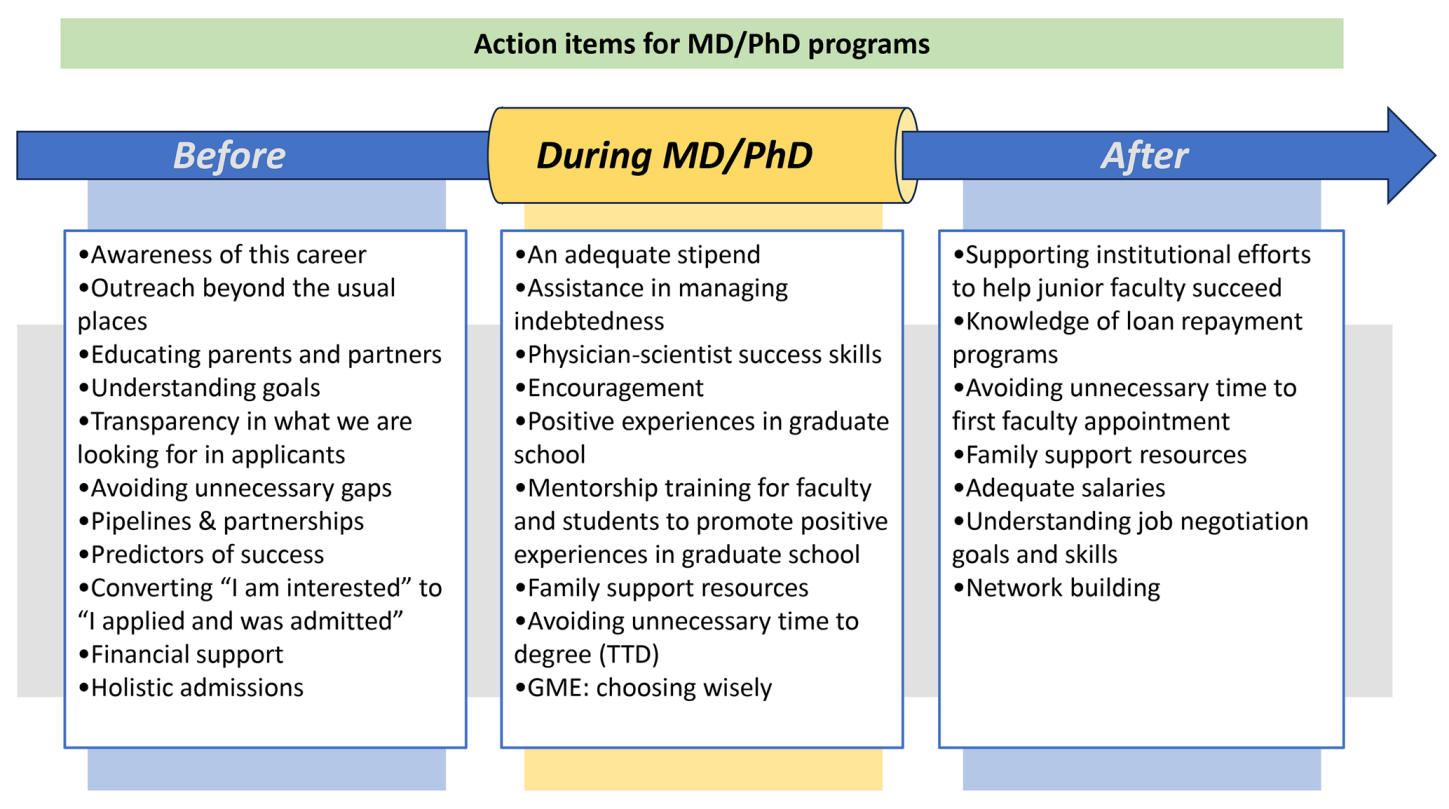
Action items for MD-PhD programs. An MD-PhD–centric view of the physician-scientist career path and opportunities to increase rates of application, matriculation, and ultimate success of diverse trainees.

Thus far, we have identified problems that contribute to underrepresentation within the physician-scientist community. As a spur for ongoing discussions, we will close with a few observations and suggestions (summarized in Figure 5). Young adults who have never heard of a physician-scientist career or an MD-PhD program are unlikely to apply. Those who have not been sufficiently coached are less likely to be admitted. Families that have no personal experience with college and/or graduate school are less likely to provide the support and encouragement needed to do both and may question the value of a career path that includes so much training time and deferred compensation. A role of the physician-scientist training community is to help heighten awareness of the rewards of a physician-scientist career, helping students and their families understand the goals of training so they can make an informed decision about the long-term value of the career.

**Figure 6:**
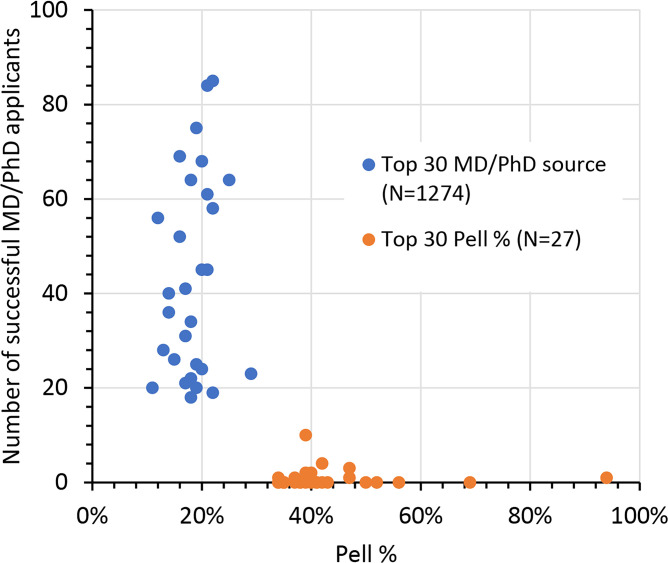
Colleges with high numbers of Pell grant recipients rarely send graduates to MD-PhD programs. The thirty colleges with the highest percentage of students with Pell grants identified from data published by the New York Times (18). The number of MD-PhD students from those colleges and the number of MD-PhD students from the top 30 source colleges was obtained from survey data collected in 2021 (15). Survey respondents were asked to indicate the college from which they obtained their undergraduate degree. 2,511 of 3,544 (71%) provided the information.

Programs can also help to grow the national applicant pool by attracting more applicants from schools that rarely send graduates to MD-PhD programs. The NIH has urged all of us to reach out to undergraduates from socioeconomically disadvantaged backgrounds, but currently most MD-PhD students come from colleges that have a low proportion of such students (15). To illustrate this point, Figure 6 compares the prevalence of Pell grants at the 30 colleges that have supplied the most MD-PhD students (15) with the colleges that have the highest prevalence of Pell grant recipients (18). There is no overlap between the two groups. Finally, pipeline programs that include MD-PhD programs in their goals can be very helpful for developing applicants who become successful trainees. Current examples include the Gateways Program at Weill Cornell (19), the Penn Access Summer Scholars Program at the Perelman School of Medicine (20), the Meyerhoff Program at the University of Maryland, Baltimore County (21, 22), and the Karsh STEM Scholars Program at Howard University. Each provides a model that can be replicated. Individual MD-PhD programs can partner with local colleges or universities to heighten awareness of the physician-scientists career path, coach applicants, and educate parents and partners.

Once admitted, the data reviewed here suggest that there are not one, but two forms of attrition on the physician-scientist career path: failing to complete the program and failing to emerge from the program still on course. Increasing the likelihood of a positive experience in graduate school through careful selection and training of mentors is very important, as is close oversight through the thesis phase of training. Programs can also help by ensuring that stipends are adequate to meet local living costs, keeping in mind that while some MD-PhD students can draw on substantial family resources, others cannot. The NIH has recently recognized that MD-PhD students may have responsibilities for others by offering help with childcare costs. Programs can help by providing additional resources and advice. Core responsibilities for MD-PhD programs already include teaching networking, team building, and survival skills. They should also include advising on the impact of clinical specialty choices on the ability to sustain a research-focused career and the long-term value of matching into residency programs that are physician-scientist friendly (7).

Finally, graduation is no longer the last responsibility of an MD-PhD program. In addition to preparing their students for a successful handoff to a physician-scientist friendly residency program, MD-PhD programs can help their trainees understand the likely job market and begin to develop a network of contacts for physician-scientist jobs in academia and elsewhere, including industry. Program leaders can also participate in the development of institutional resources intended to facilitate the fellow-to-faculty transition and help junior faculty obtain independence. Knowledge of NIH loan repayment programs can reduce indebtedness for loans that can easily date back to college.

In sum, measures taken before, during, and after MD-PhD training that foster the career success of minoritized individuals are ultimately for everyone, but they are especially important for building a diverse and inclusive physician-scientist workforce that includes more than the relatively narrow sector of economically advantaged US society that has historically sustained this career. MD-PhD programs cannot make choices for their trainees but can affect long-term outcomes by casting a wide net for applicants, providing good advice and training to those who join them, and preparing their graduates to make wise choices.

## Methods

The data set used for this study merges person-level responses from the 2015 National MD-PhD Program Outcomes Survey and AAMC data on the 6,786 individuals who completed the survey (2). Briefly, 80 MD-PhD programs, including all but 1 of the 45 programs that received NIGMS MSTP grants in 2015, participated in the outcomes survey. The programs identified 10,591 alumni and provided valid email addresses for 8,944 (84%) individuals to the AAMC data unit. Each person received an email from the current director of the program from which they graduated informing them that the program was participating in a national outcomes study and that they would receive an email on a specified date from the AAMC with an individualized active URL link to the online survey. Survey responses from 6,786 graduates (76% of 8,944) were collected on an AAMC server using Verint software. Individuals with valid email addresses who did not respond to the initial email received subsequent emails from the director of their former program and then from AAMC on 3 subsequent occasions, approximately 1, 2, and 3 months after the initial distribution of the survey. Survey data collection ended in June 2015. The authors have signed a data-sharing agreement with AAMC.

Data for race, ethnicity, and years of matriculation and graduation were obtained from AAMC databases for each survey respondent as described previously (2). Individuals were considered UIM if their self-reported race and ethnicity designation in AAMC databases indicated that they belonged to one or more of the following groups: American Indian or Alaska Native, Black or African American, Hispanic or Latino of Spanish origin, and Native Hawaiian or Other Pacific Islander. Members of these groups are designated by the NIH as underrepresented in the biomedical science workforce, regardless of whether they also identify as belonging to other race and ethnicity groups (489 respondents, of whom 241 identified as Black, 231 as Hispanic, and 27 as neither Black nor Hispanic). The non-UIM group included everyone else (6,298 individuals) who did not report belonging to one of these groups. This included 187 individuals who were listed as “non-US citizen” and for whom race and ethnicity information were not provided, and 110 individuals for whom the race and ethnicity information was designated as “unknown” (59 individuals) or “other,” with no additional specification (51 individuals). It should be noted that the AAMC changed the methods used to collect information on race and ethnicity in 2003 and again in 2014. Prior to 2003, individuals could only select one race and ethnicity response option, even if they self-identified with multiple races and ethnicities, and a separate question asked about an individual’s Hispanic origin. After 2003, individuals could select multiple response options, but the race and Hispanic origin questions remained separate. In 2014, the Hispanic origin and race response options became a single question, allowing multiple response options. As a result, the data after 2003 includes some duplicated counts of race and ethnicity responses, and the category totals may be higher than the total number of unique individuals. The AAMC updates race and ethnicity information based on the most recent entry in the American Medical College Application Service, Student Record System, Electronic Residency Application Service, Graduate Medical Education-Track, and Faculty Roster databases, which means that for some individuals, the information may have been updated. Because of these changes in methodology, comparisons of data before and after 2003 and 2014 must be done carefully. In this paper, we have chosen to present data on race and ethnicity based on the specific designations by each individual respondent. Survey response rates were essentially the same for each racial and ethnic group (2).

### Statistics.

Deidentified information released to us by the AAMC was provided in the form of a Microsoft Excel spreadsheet that included birth year, years of matriculation and graduation, sex, race, ethnicity, employment status, residency field, initial and current workplace type, academic rank, distribution of professional effort, and research awards. When indicated, comparisons were made using a 1-way ANOVA using Tukey’s multiple comparisons test to compare all data points to each other (GraphPad Prism 8).

### Study approval.

The AAMC Institutional Review Board approved the survey and the data collection and analysis processes. In the first question, participants were asked to grant permission to share their deidentified data with the authors.

## Author contributions

MHA and LFB conceived the study, analyzed the data, and wrote the manuscript. MHA and LFB contributed equally.

## Supplementary Material

Supplemental data

## Figures and Tables

**Table 1:**
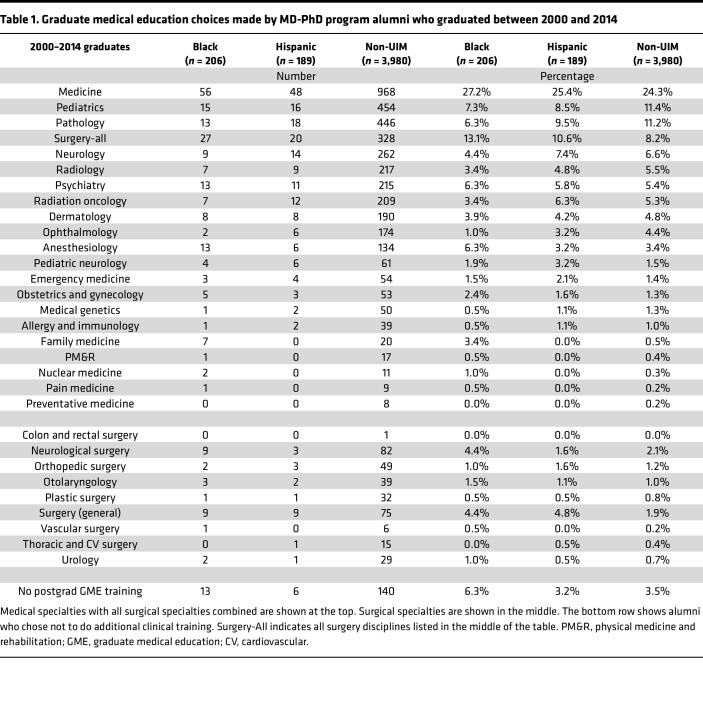
Graduate medical education choices made by MD-PhD program alumni who graduated between 2000 and 2014

## Version 1 05/08/2024

Electronic publication
