# Supplementary material for: The National MD-PhD Program Outcomes Study: career paths followed by Black and Hispanic graduates
Source: JCI Insight. 2024 May 8;9(9):e178248. doi: 10.1172/jci.insight.178248 (PMC11141911; doi:10.1172/jci.insight.178248)
Supplement: Supplemental data [file jciinsight-9-178248-s182.pdf]

Supplemental Table S1. Top 30 colleges for producing MD/PhD program students*

Rank	College	# into MD/PhD	Freshman Pell share
1	Harvard	85	22%
2	Yale	84	21%
3	University of Pennsylvania	75	19%
4	Washington University in St. Louis	69	16%
5	Johns Hopkins University	68	20%
6	Cornell	64	18%
7	UC Berkeley	64	25%
8	Columbia	61	21%
9	UCLA	58	22%
10	Duke	56	12%
11	University of Michigan	52	16%
12	MIT	45	21%
13	Stanford University	45	20%
14	University of Wisconsin - Madison	41	17%
15	University of Chicago	40	14%
16	Brown	36	14%
17	Princeton University	34	18%
18	Ohio State University	31	17%
19	University of Virginia	28	13%
20	University of Maryland College Park	26	15%
21	Emory	25	19%
22	Northwestern University	24	20%
23	University of Texas at Austin	23	29%
24	Vanderbilt University	22	18%
25	Rice University	21	17%
26	Tufts University	20	11%
27	University of Maryland Baltimore Co.	20	ND
28	University of Rochester	20	19%
29	University of Florida	19	22%
30	New York University	18	18%
		total	average
		1274	18%

*The data on matriculants into MD/PhD programs are from reference (15). The data on percentage of freshman who were Pell grant recipients are from reference (18).

Supplemental Table S2. Top 30 colleges for Pell grant share*

Rank	College	Freshman Pell share	# into MD/PhD
1	Berea College	94%	1
2	Salem College	69%	0
3	CUNY Baruch College	56%	0
4	College of Mount St. Vincent	52%	0
5	College of the Ozarks	50%	0
6	Agnes Scott College	47%	1
7	Florida International University	47%	3
8	Pacific Union College	43%	0
9	Houghton College	42%	0
10	University of New Mexico	42%	4
11	Brescia University	41%	0
12	Stockton University	41%	0
13	Earlham College	40%	0
14	Lake Forest College	40%	0
15	Millsaps College	40%	2
16	Christian Brothers University	39%	0
17	Hampshire College	39%	1
18	Lyon College	39%	2
19	Stony Brook University	39%	1
20	University of California, Irvine	39%	10
21	King University	38%	0
22	Loyola University New Orleans	38%	0
23	Beloit College	37%	0
24	University of the Pacific	37%	1
25	Knox College	35%	0
26	New College of Florida	35%	0
27	Willamette University	35%	0
28	Mount St. Mary's University	34%	1
29	New Mexico Inst. of Mining and Tech.	34%	0
30	Southwestern University	34%	0
		average	total
		43%	27

*The data on matriculants into MD/PhD programs are from reference (15). The data on percentage of freshman who were Pell grant recipients are from reference (18).
